# Animal Models of IgE Anaphylaxis

**DOI:** 10.3390/biology12070931

**Published:** 2023-06-29

**Authors:** Aurélie Gouel-Chéron, Alice Dejoux, Emma Lamanna, Pierre Bruhns

**Affiliations:** 1 Université Paris Cité, 75010 Paris, France; 2 Anaesthesiology and Critical Care Medicine Department, DMU Parabol, Bichat-Claude Bernard Hospital, AP-HP, 75018 Paris, France; 3 Institut Pasteur, Université de Paris Cité, INSERM UMR1222, Antibodies in Therapy and Pathology, 75015 Paris, France; 4 Sorbonne Université, Collège Doctoral, 75005 Paris, France; 5 Neovacs SA, 92150 Suresnes, France

**Keywords:** allergy, anaphylaxis, IgE, animal models

## Abstract

**Simple Summary:**

Anaphylaxis is the most severe form of allergic reactions and can be life-threatening. It is very difficult to study the mechanisms underlying anaphylaxis in humans since these events are rare and often lethal. Therefore, animal models have been established. Mice and rats are mostly used since their biological parameters, such as temperature drop, behavioral changes, and blood or cell biomarkers, can be easily measured in the laboratory. These animals can also be genetically modified to express human proteins and cell functions. Different animal models have been established to replicate as closely as possible the natural route of sensitization to the allergen and to trigger anaphylaxis in animals. These animal models have deepened our knowledge on human anaphylaxis with certain limitations, as discussed in this review.

**Abstract:**

Allergies and atopy have emerged as significant public health concerns, with a progressively increasing incidence over the last two decades. Anaphylaxis is the most severe form of allergic reactions, characterized by a rapid onset and potentially fatal outcome, even in healthy individuals. Due to the unpredictable nature and potential lethality of anaphylaxis and the wide range of allergens involved, clinical studies in human patients have proven to be challenging. Diagnosis is further complicated by the lack of reliable laboratory biomarkers to confirm clinical suspicion. Thus, animal models have been developed to replicate human anaphylaxis and explore its pathophysiology. Whereas results obtained from animal models may not always be directly translatable to humans, they serve as a foundation for understanding the underlying mechanisms. Animal models are an essential tool for investigating new biomarkers that could be incorporated into the allergy workup for patients, as well as for the development of novel treatments. Two primary pathways have been described in animals and humans: classic, predominantly involving IgE and histamine, and alternative, reliant on IgG and the platelet-activating factor. This review will focus essentially on the former and aims to describe the most utilized IgE-mediated anaphylaxis animal models, including their respective advantages and limitations.

## 1. Introduction

Allergies and atopy have emerged as significant public health concerns with a growing incidence over the last two decades, for reasons not yet entirely understood [1]. Anaphylaxis, the most severe form of acute hypersensitivity reaction, has been defined as a “serious allergic reaction that is rapid in onset and may result in death” [2]. It can be fatal, even in healthy individuals, with food and drugs being the most widely recognized culprit agents [3]. There are different definitions of anaphylaxis in the literature: some require a systemic and generalized reaction, whereas other definitions require symptoms in one organ system [4]. Typical symptoms (also called phenotype) of anaphylaxis include skin rash, respiratory symptoms with bronchospasm, cardiovascular symptoms with arterial hypotension, tachy/bradycardia and cardiac arrest in the most severe form, and gastrointestinal difficulties [5]. According to the World Allergy Organization, anaphylaxis is diagnosed when one of the following criteria is fulfilled: (1) an acute onset of an illness with the involvement of the skin or mucosal tissues, or (2) an acute onset of hypotension or bronchospasm even in the absence of skin involvement.

In human patients, studies of anaphylaxis are challenging because of the rapid onset and potential lethality of the reaction. For obvious ethical reasons, inducing anaphylaxis in humans is not acceptable. Most of the time, the reaction takes place outside the hospital, making it difficult to study the early stage of the reaction. Epinephrine is the predominant treatment against anaphylaxis since it counteracts the physiologic changes induced by anaphylaxis through the activation of adrenergic receptors by inducing, among other effects, vasoconstriction and bronchodilation. Because of this context and the lethality of the reaction, randomized control trials analyzing epinephrine compared to another treatment, such as methylene blue, vasopressin, or norepinephrine, would not be acceptable without strong animal evidence of efficacy. Most data in humans have so far relied on clinical case series [6,7]. Additionally, the type and quantity of allergens, as well as the route of exposure, may vary greatly between patients. Symptoms can occur within seconds to a few hours after allergen exposure and can affect multiple organ systems, although not all may be affected in a single allergic reaction. Early intervention is essential, and it has been identified as a positive prognostic factor [8]. The lack of reliable biomarkers to confirm the clinical prognosis and the difficulties in diagnosis further impede research into anaphylaxis in the clinic.

Due to these challenges, animal models have been established to reproduce as faithfully as possible the pathophysiology of human anaphylaxis [9]. Such tools include genetically modified strains that can express humanized proteins (i.e., receptors and ligands) or suppress the expression of murine genes. Although findings obtained from animal models may not completely replicate human anaphylaxis, they provide a framework for comprehending the mechanisms at work [10]. Furthermore, they are an invaluable tool for exploring potential new biomarkers that could be included in the allergy work-up for patients and new treatments.

The classical pathway of anaphylaxis involves immunoglobulin (Ig) E antibodies, but other alternative pathways have been described in humans. The non-IgE mediated pathways include the formation of IgG/antigen complexes that can activate effector cells such as neutrophils, macrophages, and monocytes [11]. This review aims to outline the most used IgE-mediated anaphylaxis animal models and their respective advantages and limitations.

## 2. Physiopathology

### 2.1. Antibodies in IgE-Mediated Anaphylaxis

Two independent mechanisms have been described in animal models of anaphylaxis, each of them involving antibodies of a distinct isotype (IgE or IgG); the first of these, referred to as the classical pathway, is the subject of this review [11]. Ig is a symmetrical protein composed of two identical heavy and light chains that possesses a variable and a constant region. The amino acids forming the variable region are involved in antigen binding and define the specificity of the antibody for a unique antigen. The constant region is responsible for mediating effector functions by binding to receptors expressed on the cell surface. This determines the underlying mechanisms activated in response to the presence of antibodies recognizing the antigen. Such mechanisms can include cell activation, endocytosis, release of inflammatory mediators, and complement activation. The heavy-chain constant region also determines the antibody isotype: IgG, IgA, IgM, IgD, or IgE, each possessing a distinct role [12].

The binding of Ig to antibody Fc receptors (FcR) expressed on the surface of effector cells triggers a biological response that can include activating or inhibitory signals. The formation of an antibody–antigen complex is necessary to trigger the cellular response by causing receptor aggregation or cross-linking. FcRs possess different affinities for their respective Ig isotypes, which can vary from one species to another [13]. Most FcRs, however, are of low affinity and only retain Ig if present as an immune complex, opsonized on a surface, or aggregated. Rare high-affinity receptors exist that can bind and retain Ig, such as the IgE receptor FcεRI. In humans, FcεRI and FcεRII (CD23) are IgE receptors, FcγRs are IgG receptors, and FcαRI (CD89) is an IgA receptor. Mice do not express FcαRI, and most of their IgG receptors bind IgE with low affinity (inhibitory mouse FcγRIIB and activating mouse FcγRIII and FcγRIV) [14,15]. FcεRI, the high-affinity IgE receptor, is composed of α, β, and γ subunits (αβγ_2_ tetramers) in human and mouse mast cells and basophils, whereas it is composed of only α and γ subunits (αγ_2_ trimers) in human macrophages and Langerhans cells [15]. These differences in the cell expression pattern might alter the translation of mouse experimental results to humans. The β subunit enhances signaling through FcεRI but is not required for binding; indeed, it has been shown that the α subunit of the hIgE receptor is sufficient for high-affinity IgE binding [16].

### 2.2. Mechanisms of IgE-Mediated Anaphylaxis

**Figure 1:**
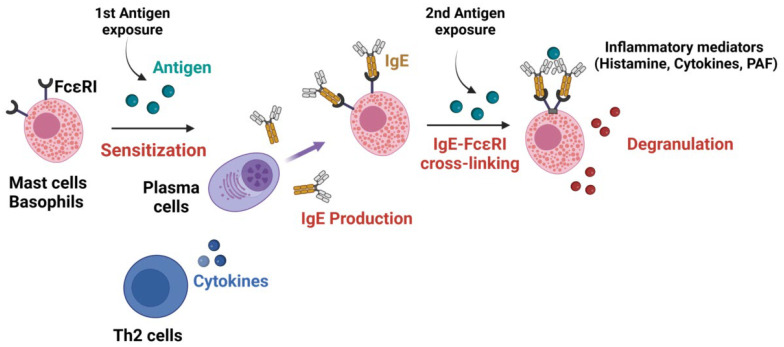
Pathway of IgE-mediated anaphylaxis. A first antigen exposure is required for specific IgE synthesis by plasma cells in a Th2 environment. The second antigen exposure enables the cross-linking of FcεRI by the binding of the antigen on the specific sIgE/FcεRI complex. The sIgE/FcεRI aggregation triggers mast cell and basophil degranulation, which releases inflammatory mediators responsible for the clinical symptoms of anaphylaxis.

Although other mechanisms have been described, the classical pathway of anaphylaxis involves IgE antibodies bound to their high-affinity receptors (FcεRI), which are mainly expressed on the surface of mast cells in tissues and basophils in circulation. This pathway requires a two-step process. First the sensitization phase: pre-exposure to the antigen that leads to the synthesis of a specific IgE (sIgE) that binds on the surface of the effector cells in a T-cell helper type 2 (Th2) environment. Activated Th2 lymphocytes secrete cytokines IL-4, IL-5, and IL-13, which induce IgE class switch recombination in B cells and their differentiation into IgE-secreting plasma cells. This secreted IgE binds to FcεRI on mast cells forming IgE/FcεRI complexes. The second phase occurs following a second antigen exposure, during which antigens bind to at least two IgE/FcεRI complexes enabling the cross-linking of the receptor and the induction of signal transduction by the γ and β chains. This sIgE/FcεRI aggregation triggers the degranulation of pre-stored and de novo synthesized inflammatory mediators such as histamine (primarily responsible for the development of the reaction), tryptase, platelet-activating factor (PAF), prostaglandin D2, and leukotrienes, leading to clinical signs of anaphylaxis (Figure 1) [17]. Mast cells are key players in allergic disease and have been recognized as the main cell responsible for anaphylaxis events for more than 50 years [18]. Indeed, their activation by the antigen-induced IgE crosslinking of FcεRI induces a feed-back loop stimulating mast cell survival and, in B cells, a mechanism of immunoglobulin class switch to IgE, further promoting Th2-type immunity [19]. Mast cell mediators can be released by either sIgE/FcεRI aggregation in the presence of the specific antigen or by IgE-independent degranulation. Indeed, some drugs can directly trigger mast cell degranulation in vitro via the activation of the mast cell receptor ‘Mas-related G protein-coupled receptor X2’ (MRGPRX2) [20], although no formal proof has been reported *in vivo* in humans so far. Finally, human cardiac mast cells are also suspected to be involved in cardiac anaphylaxis phenotypes, such as the type 1 Kounis syndrome, which is characterized by vasospasm in healthy coronary arteries [21].

Whereas the nature of the allergen and the route of sensitization are usually well known in food or venom anaphylaxis, this is not usually the case for drug allergies, since some molecules are non-immunogenic and drug-induced anaphylaxis occurs frequently upon the first exposure to the patient. The mechanism of drug sensitization has been the subject of intense research in recent years [22]. The leading theory is that some drugs can form a complex with a carrier protein (the hapten theory) that will be recognized as a neo-antigen.

## 3. Anaphylaxis Monitoring in Animal Models

The use of animals in research requires approval from an ethical committee and compliance with the Guiding Principles for Research Involving Animals, regardless of the species involved. Whereas animal models have been predominantly established in mice and rats, reports have also been published for other species such as rabbits [8], guinea pigs [23], dogs [24], sheep [25], pigs, and swine [26]. These have been largely described elsewhere and are not the subject of this review [27,28].

Anaphylactic reactions can include a large range of symptoms in humans, such as arterial hypotension, vasodilation, bronchoconstriction, erythema, and arrhythmia as previously described [4]. To model anaphylaxis in animals, the consequences of the induced reaction on the hemodynamic and respiratory system must be measured. In mouse models, invasive monitoring can present challenges because of the small size of the animal. Although feasible, it requires specific equipment, and surgery which is time-consuming to setup and therefore not often used. To replace this approach, clinical noninvasive tools are thus required. Hypothermia, measured by rectal temperature, is widely used to evaluate anaphylaxis severity in mice, as it is considered a surrogate marker of cardiac output. Other monitoring tools can include a quantitative mouse activity scale (3, normal activity; 2, slow movement after prodding; 1, no movement in response to prodding; and 0, inability to right itself after being turned on its side) and diarrhea occurrence, which is thought to positively correlate with levels of intestinal mast cells [29]. To the best of our knowledge, most of the anaphylaxis mouse models reported so far relied on these measures to assess anaphylaxis.

As opposed to mice, all anaphylaxis models on rats have used invasive measurements to assess the impacts of anaphylaxis on the respiratory and hemodynamic system. As rats are larger in size compared to mice, certain procedures are easier to perform, such as tracheal intubation or tracheotomy; artery and vein line placement (femoral, jugular, or carotid); and the insertion of tissue oxygen pressure (PtiO2) and microdialysis probes. These procedures enable the monitoring of mean arterial pressure, blood flow velocity, vascular peripheral resistance, and muscular interstitial lactate concentration. They also allow biological sampling such as arterial blood gases and hematologic measurements. A precise evaluation of the decreased vascular resistance, fluid loss, and hemodynamic impairment induced by a reaction can be performed with these monitoring devices (Table 1). Respiratory resistance and elastance, microvascular leakage in the airways [30], and extensive cerebral monitoring [31] can also be more easily performed in rats, including measurements of cerebral cortical blood flow, carotid artery blood flow, cerebral oxygen partial pressure, cerebral interstitial lactate/pyruvate ratio, and PtiO2 via electrode insertion after craniotomy into the right cerebral cortex [32,33,34]. The objectives are to evaluate the consequences of the hemodynamic failure induced by anaphylaxis on the cerebral oxygenation (Table 1). Hemodynamic measurements (micro- and macro-circulation), including cardiac output, skeletal muscular oxygen partial pressure, skeletal muscular interstitial lactate/pyruvate ratio, and PtiO2, can also be extended using perivascular ultrasonic flow probes placed around the upper abdominal aorta or by inserting an electrode into muscles, among other techniques [32]. Left-ventricular function can be assessed by inserting a catheter into the left ventricle to measure direct pressure. Similarly, as performed in humans, cardiac echography enables left-ventricular end-diastolic and systolic diameter measurements, allowing for the calculation of the left-ventricular shortening fraction [35]. Although these measures have been reported in mice, they have never been assessed in anaphylaxis models [36].

Acute hemoconcentration, as assessed by an increase in the hemoglobin concentration in the absence of acute diuresis, has been suggested as a highly indicative marker of anaphylaxis occurrence and vascular leak. Hematocrit measurement and, consequently, the correction of hemoconcentration can attest to the efficacy of volume expansion and have been suggested as a monitoring tool in animal studies [37]. However, performing blood draws on a mouse during an anaphylactic reaction is hardly feasible, if not impossible, as the blood pressure is too low. However, animals are often sacrificed at the end of the anaphylaxis experiments, allowing for blood sampling and tissue collection for biomarker measurement, cytometry, and histological analyses [38,39].

In most mouse anaphylaxis models reported so far, clinical surrogate measurements have been used (rectal temperature, behavior scale), whereas the use of larger animals, such as rats, enables the measurement of particular outcomes such as hemodynamic changes, particularly in assessing treatment efficacy and its effects on cerebral oxygenation, cardiac output, and vascular leakage [40,41].

## 4. Animal Models

In immunization procedures, the use of adjuvants is imperative to bypass the phenomenon of oral tolerance to proteins observed in most animal species. The goal is to induce clinical responses that closely resemble IgE-mediated food and drug allergies in humans. The extent of sensitization depends on various factors, including antigen concentration, administration route and duration, animal age and strain, Th1/Th2 polarization (which is more distinct in mice compared to humans), and use of adjuvants.

### 4.1. Sensitization Routes

Sensitization success varies considerably according to the route of administration, dose, and frequency of injections. Various routes of immunization can be used to induce sensitization, including oral gavage; epicutaneous, subcutaneous, or intradermal exposure; and intraperitoneal or intravenous injections.

It is generally accepted that among adjuvants, alum favors the production of IgG1 and IgE antibodies in wild-type mice, whereas Complete Freund’s Adjuvant (CFA) favors IgG2 antibodies, even if mice with different genetic backgrounds can display different responses to immunization with these adjuvants [42]. In both cases, however, IgG1 antibodies are the most abundant and IgE the least abundant. 

The route of sensitization chosen should depend on the allergen used and the human disease studied. In food-driven anaphylaxis, oral sensitization is predominantly used [43]. This model emphasizes the effect of digestion on sensitization and stimulate the interactions of the epithelia with the allergens. Indeed, it has been shown that the intestinal epithelial barrier regulates protein antigen passage and guides mucosal immune responses. One protocol example is intragastric feeding once a week for 7 weeks with the allergen and cholera toxin using a ball-ended feeding needle [44]. It was established in a mouse model of anaphylaxis sensitized with different doses of peanut protein via intragastric gavage that lower doses of peanut protein induced the highest IgE levels and a more severe anaphylactic reaction [45]. This suggested that lower doses of allergen could induce a stronger sensitization compared to higher allergen doses. 

Sensitization via the skin can be performed epicutaneously or subcutanesouly. Epicutaneous sensitization is performed on abraded skin. There are various methods to perform this type of sensitization: (1) calcipotriol application on a specific skin area for 14 consecutive days, which inhibits keratinocyte proliferation and generates an atopic-dermatitis-like skin lesion [46,47]; (2) the application of the allergen directly on a shaved skin area under a transparent bio-occlusive dressing for 7 days, with the same treatment being applied at the same site for a total of three 1-week exposures, separated by a 14-day period [48,49,50,51]; and (3) the induction of a skin injury to mimic the mechanical injury caused by scratching. This route of sensitization is very useful to mimic skin-driven allergies but is also justified for other antigens such as peanut proteins. Indeed, a human study demonstrated that peanut sensitization occurs as a result of environmental household exposures rather than maternal peanut consumption during pregnancy [52]. Most animal studies support the evidence that subcutaneous sensitization is the most effective in mouse asthma models. In a study comparing intraperitoneal, subcutaneous, and aerosol sensitization using the major birch pollen allergen Bet v 1, the subcutaneous route elicited higher IgE levels and the preferential production of Th2 cytokines in spleen cells [53].

A fourth sensitization route relies on intraperitoneal injections. Several strategies can be mixed. Jonsson et al. [54] reported three intraperitoneal injections on days 0, 14, and 28 using BSA as the antigen either with CFA for the first injection followed twice by Incomplete Freund’s Adjuvant; or three times with alum; or three times with alum plus Pertussis toxin. The challenge performed 10 days after the last immunization induced a strong anaphylaxis reaction in mice with high lethality [54]. This route has been shown to be more efficient than other routes of sensitization in some animal studies. For instance, Liu et al. [55] sensitized mice with wheat gluten via intraperitoneal, transdermal, and oral gavage sensitization routes. They showed that all three methods could induce allergic symptoms (increased serum antibodies, Th2 secretion, and inflammatory factors). Nonetheless, levels of serum antibodies were higher in mice sensitized intraperitoneally, and the bacterial species diversity in the intestinal flora was more significantly decreased in this group of mice as well [55]. The comparison of oral, intraperitoneal, and subcutaneous sensitization in ovalbumin (OVA) anaphylaxis led to the generation of OVA IgE-epitopes in all models, with more binding epitopes in intraperitoneal compared to oral immunization [56]. However, the answer is not straight forward, as epicutaneous sensitization can elicit a stronger immune response than the intraperitoneal route in some cases. Several mouse studies supported this hypothesis, with higher OVA-specific serum IgE and IgG antibody responses after epicutaneous compared to intraperitoneal sensitization. They suggested that epicutaneous sensitization may be more prone to inducing a Th2 response [48].

Combining sensitization routes could also be of interest, as the combination of intraperitoneal and epicutaneous sensitization has been shown to be more effective than epicutaneous alone in a mouse model of skin disease (atopic dermatitis) [57]. 

Intradermal sensitization is mostly used for passive cutaneous anaphylaxis (PCA) (Section 4.2) by injecting the antigen under the epidermis. This route of injection has the longest absorption time and the advantage of being easily visualized. 

Intravenous allergen injection in mouse is mostly used for antigen challenge after a prior sensitization. This route of allergen administration is useful to closely mimic drug-induced anaphylactic reactions in humans, which are often triggered by the intravenous injection of high doses of drugs. However, it might be used for sensitization when the mice need to be passively sensitized with an antibody before the antigen challenge during passive systemic anaphylaxis (PSA) (Section 4.3). The antibody administered intravenously can directly bind the receptors at the surface of effector cells prior to allergen exposure. 

As there has been a lot of variation in the choice of route, immunization frequency, and the concentration of antigen sensitization across different studies, it is crucial to carefully optimize these parameters when implementing animal models of anaphylaxis. 

### 4.2. Passive Cutaneous Anaphylaxis

PCA was among the earliest models described, and, consequently, it played a significant role in the comprehension of anaphylaxis pathophysiology. The model involves the intradermal injection of allergen-specific IgE antibodies into mice, followed by intravenous challenge with the corresponding allergen [58,59]. Mouse IgE monoclonal antibodies (mAbs) can be used in mice expressing endogenous FcεRI (mFcεRI), whereas human IgE mAbs or serum from an allergic patient can be used in transgenic mice expressing human FcεRI (hFcεRI). 

Evans Blue dye can be administered with or closely following antigen challenge to determine changes in vascular permeability as demonstrated by the leakage of the dye into the reaction site. This method provides a visual representation of the hypersensitivity reaction, as well as a quantitative result when extracting postmortem the dye from the skin and measuring its optical density.

### 4.3. Passive Systemic Anaphylaxis

Passive systemic anaphylaxis (PSA) induced by IgE is an established model for the study of anaphylaxis. The model involves the systemic injection of specific IgE antibodies in mice, followed 24–48 h later by challenge with the corresponding antigen. Passive sensitization can be performed with monoclonal antibodies (mouse or human if mouse express human FcεRI) or with a pool of immunized human or mouse sera [60,61]. Anaphylactic shock develops within minutes and can be assessed as described in Section 3, most practically by recording hypothermia. In the case of oral administration, mice should fast for 3 to 4 h prior induction. This model has been instrumental in the understanding of the IgE-dependent classical pathway mediated by mast cells and the release of histamine [62].

Dombrowicz et al. [59]. demonstrated that mice deficient in mFcεRI were protected from developing anaphylaxis, showing that FcεRI is necessary for the induction of IgE-mediated anaphylaxis. In addition, IgE-induced PSA was abrogated in mast-cell-deficient W/W^v^ mice [63] and after the injection of anti-IgE mAbs [64].

The role of histamine and mast cells in PSA has been demonstrated in studies with mice lacking histamine (using histidine decarboxylase-deficient mice) or mice injected with histamine receptor antagonists to prevent anaphylaxis [65]. All the studies performed using this model demonstrated the importance of mFcεRI and mast cells in IgE-induced PSA, as they release histamine following the crosslinking of IgE molecules bound to the mast cell surface via mFcεRI.

### 4.4. Active Systemic Anaphylaxis

The active systemic anaphylaxis (ASA) model differs from the PSA model as it involves sensitization to a specific allergen using the allergen itself rather than bypassing the immunization phase by providing passively specific antibodies against the allergen. The purpose of this process is to mimic the sensitization process that occurs in humans prior to IgE- or IgG-dependent anaphylaxis and requires antibodies to be produced by the host. 

Although ASA and PSA present similar symptoms, a higher mortality rate is observed in ASA, and the results obtained from these models do not always correlate [66]. ASA is frequently used in research on alternative pathways of anaphylaxis, as it highlights the indispensable role of IgG and IgG receptors in ASA and the downstream production of PAF, which mediates the physiological symptoms of ASA [54,67].

### 4.5. Intestinal Models of Anaphylaxis

Several animal models have been described to study food-allergy-induced diarrhea and shock. In the model, mice were sensitized via intraperitoneal injection with ovalbumin emulsified in alum, twice and two weeks apart [68,69]. In the second model, mice were sensitized through the ingestion of an allergen, such as peanut extract, with cholera toxin as a mucosal adjuvant [45]. In the third model, mice were sensitized through epicutaneous exposure to hazelnut extract, where the allergen was applied to the mouse’s clipped back skin and covered with a bandage for three days [70,71].

Challenges with the appropriate allergen were performed through intra-gastric gavage after depriving mice of food for 3–4 h. Interestingly, the ovalbumin model only induced diarrhea, whereas the peanut and hazelnut models induced both diarrhea and shock. The reason for this difference was not immediately clear and may be related to the allergen nature, molecular structure, and dose, which could lead to differences in allergen absorption or activate different pathways.

These studies have played a crucial part in elucidating the role of the classical mast-cell- and IgE-mediated mechanisms in allergic manifestations with gastrointestinal symptoms, including diarrhea, in response to food allergens. A mouse model of oral antigen-induced anaphylaxis with intestinal and systemic symptoms revealed that diarrhea and hypothermia were mitigated in mFcεRI-deficient mice following challenge compared to controls [29]. Intestinal mast cell levels were also found to be correlated with the severity of these symptoms.

### 4.6. Transgenic Mice and Humanized Mouse Models

Transgenic mouse models have been developed to investigate the involvement of specific cytokines, such as IL-4 and IL-13, in anaphylaxis [72], as well as the roles of various effector cells, antibodies, and receptors. For example, mice deficient in cytokine IL-9 and its receptor were used to study the role of IL-9 in intestinal anaphylaxis [73], whereas mice deficient in the IL-33 receptor (ST2) were used to evaluate the role of IL-33 in IgE production and mast cell degranulation [74]. Mice deficient in CC chemokine receptor 4 (CCR4), which is expressed on skin-homing T cells, showed the involvement of T-cells during oral sensitization [44], whereas platelet endothelial cell adhesion molecule-1-deficient mice demonstrated its properties as a counter regulatory mechanism in allergic disease susceptibility and severity [75].

To precisely analyze the effect of a single human effector cell, antibody, or receptor, several “humanized” models of anaphylaxis have been developed and can be used in PCA, PSA, ASA, and intestinal anaphylaxis models. They were established to study anaphylaxis in a manner that more closely mimics human physiology. These models include mice expressing hFcεRI and mice deficient in various or all FcRs.

Mice expressing hFcεRI instead of mFcεRI display a similar cell expression profile of hFcεRI to humans [76]. Whereas mouse IgE can bind to human FcεRI, human IgE cannot bind to mouse FcεRI. To investigate the role of hFcεRI in anaphylaxis, these transgenic mice were used in PSA [59] and PCA [58] models. mIgEs are able to bind not only to the high-affinity IgE-receptor mFcεRI but also to low-affinity IgG-receptors, such as mFcγRIIB and mFcγRIII. The use of knock-out mice has revealed that IgE-mediated anaphylaxis can be modulated by IgG receptors, such as mFcγRIIB (the IgG inhibitory receptor) (with an enhanced reaction in mFcγRIIB-deficient mice) and mFcγRIII (an IgG activating receptor, leading to an attenuated reaction in mFcγRIII-deficient mice). Both receptors are present on mast cells and can act as regulators of IgE-mediated anaphylaxis in mice [77]. Transgenic mice with a gain-of-function mutation in the immunoreceptor tyrosine-based inhibitory motif (ITIM) at position 709 (Y→F) of the IL-4 receptor (IL-4Rα) displayed elevated IgE levels and increased susceptibility to allergen-induced airway inflammation, similar to atopic individuals. In these mice, the amplification of IL-4Rα signals facilitated allergic sensitization to ingested antigens and drove IgE-dependent anaphylactic responses [78,79,80]. These mice could also be useful in experiment setup in which robust allergic sensitization cannot be achieved due to tolerizing effects. Finally, highly immunodeficient NOD-scid γc^−/−^ (NSG) mice could be engrafted with human hematopoietic stem cells to study hIgE-mediated reactions and were used for PCA and ASA peanut allergic models [81,82,83]. Following engraftment, NSG mice developed large numbers of human mast cells in the peritoneal cavity and peripheral tissues, were able to produce hIgEs and hIgGs in response to sensitization, and could develop anaphylaxis after challenge.

Other mouse models have been developed to investigate the role of IgG in anaphylaxis and the importance of the IgG pathway compared to the IgE-mediated pathway. Indeed, the generation of an anti-allergen IgE response requires an initial IgG response and the switching of IgG B cells to IgE B cells. Anti-allergen IgG may thus positively or negatively regulate the IgE pathway in anaphylaxis and hence anaphylaxis symptoms and severity. Indeed, mice deficient in both IgE receptors, FcεRI and FcεRII (CD23), developed ASA similarly to wild-type mice. However, mice deficient in all activating FcRs (IgG and IgE receptors) were protected from ASA [54], suggesting that mouse IgG receptors are sufficient to induce anaphylaxis. The same was demonstrated using transgenic mice for human IgG receptors: in mice deficient in all endogenous mouse FcRs, the expression of hFcγRIIA was found to be sufficient to induce fatal ASA [84], and the expression of hFcγRI was sufficient to induce mild ASA [85]. More recent mouse models expressing all human low-affinity IgG receptors as knock-ins demonstrated the predominance of hFcγRIIA in PSA (using human IgG antibodies) and ASA (based on endogenous mouse IgG production) models, leading to neutrophil activation and PAF release [86]. Mice expressing all hFcγRs as transgenes showed that hFcγRs can induce PSA when injected with heat-aggregated IVIG [87].

Overall, these models offer a powerful set of tools for investigating potential new human treatments for anaphylaxis, such as histamine receptor 1 antagonists, omalizumab (an anti-IgE-capture antibody) [82], anti-FcγRIIA blocking antibodies, and PAF receptor antagonists [86]. More models are currently being developed that aim to reconstitute the expression of both human IgE receptors, hFcεRI and hFcεRII, and the production of human IgE for the study of the human IgE pathway in in vivo models.

## 5. Animal Strain Influence

The use of mouse models to study anaphylaxis is complicated by the differences in responses to sensitization and challenge exhibited by mice of different genetic backgrounds. Strains such as BALB/c, DBA/2, C3H/HeJ, BDF-1, A/J, 129S5, and C57BL/6 have been employed. BALB/c strain mice are the most commonly used, presumably because BALB/c strain mice favor Th2 over Th1 responses, and because of their ability to develop airway responsiveness during lung challenge. However, they do not systematically develop anaphylaxis compared to other mouse strains. Indeed, in a peanut allergy model using intramuscular immunization with DNA encoding for an allergen, C3H/HeSn mice displayed anaphylaxis after challenge at weeks 3 and 5 after immunization, whereas AKR/J and BALB/c mice did not. All three strains displayed increased specific IgG2a, but increased specific IgG1 or IgE was only detected in C3H mice [88]. Similarly, in an active sensitization model using intraperitoneal injections of peanut extract or purified Ara h 2, C3H/HenHsd mice, but not BALB/C and C57BL/6 mice, experienced clinical symptoms of anaphylaxis and temperature loss following antigen challenge [61,78]. Furthermore, 129S5 mice also demonstrated an increased susceptibility to anaphylaxis compared to BALB/c mice [89]. In a recent study in our laboratory, we found that BALB/c and C57BL/6 mice produced different subclasses of IgG in response to the same allergen in alum, leading to the retention of the allergen in the lungs of BALB/c, but not C57BL/6, mice during anaphylaxis challenge [42]. 

Regarding rats, the Brown Norway rat is a high-IgE-responder strain that does not require an adjuvant for sensitization to an allergen and has been widely used. Other strains such as Wistar, Hooded Lister, and Piebald Virol Glaxo have also been studied, but their inability to produce antigen-specific IgE make them less suitable for anaphylaxis models [90,91,92]. 

Careful animal strain selection is mandatory to adapt the strain to the sensitization and challenge method (or vice-versa) and should be considered in the context of results obtained using other animal strains to draw conclusions on allergen-induced reactions in humans.

## 6. Limits of Animal Models in Anaphylaxis

Rodents, particularly mice and rats, are widely used in laboratory models due to their availability; low cost; and well-understood metabolism, physiology, and biochemistry pathways. However, their distinct antibody and receptor profiles compared to humans must be considered when interpreting study results and extrapolating findings to human patients. Whereas these species share some pathophysiological similarities, such as in sepsis models [93], the time-course of anaphylaxis development, the lack of supportive therapeutic interventions, and the relatively allergen-free environment of laboratory facilities limit the translation of animal models to human anaphylaxis. Indeed, human medical intervention usually takes place minutes (in the peri-operative setting) to hours (when anaphylaxis occurs outside the hospital or following oral intake) after the reaction, whereas animal models focus on seconds to minutes after induction. Humans develop in an environment full of allergens, whereas animals are only sensitized to one defined antigen, which might modify the immune/antibody response in animals compared to humans. This will undeniably affect biomarker dynamics and therapeutic efficacy. Animal models typically use young adult animals with similar genetic backgrounds, age, gender, weight, and nutritional status, whereas human anaphylaxis occurs in patients with diverse ethnicities/genetics, food diets, gender, age, weight, and potential ongoing medical treatments and comorbidities. Moreover, most animal models rely on high antigen quantities for immunization and challenge, which may involve different pathways compared to human pathways leading to anaphylaxis [67]. The immune systems of animals and humans differ in antibody subclasses and the distribution of antibody receptors. As discussed previously, FcɛRI, for example, is more widely expressed in human subjects, including on macrophages and dendritic cells, whereas in mice, it is only expressed on mast cells and basophils [66].

Therefore, translating animal model findings to human clinical practice is highly challenging. Despite these limitations, animal models remain valuable tools to enhance our understanding of anaphylaxis pathophysiology and treatment, as long as their advantages and limitations are considered in the study design and result interpretation.

## 7. Perspectives

One missing aspect in anaphylaxis animal models is the translation of results obtained in small rodents to larger mammals that are more relevant in terms of physiology to humans. As illustrated in this review, published mouse and rat models have not focused on the same aspects. Whereas rat models have mostly been used so far to investigate treatment efficacy and physiological changes after anaphylaxis induction and treatment, mouse models have mostly focused on sensitization and the cellular and molecular physiopathology of anaphylaxis. Small animals are easier and cheaper to manage and reproduce and therefore represent a useful tool for developing models of anaphylaxis. However, large animals might better reproduce human physiopathology and might be considered for result validation before translation into clinical practice. An ideal animal model should display the same level of tolerance to antigens as humans, with a similar allergenicity profile; require the same amount of allergen to induce sensitization via the same route and duration of exposure; and present the same Th1/Th2 polarization and the same antibody profile as humans. It should also allow the realization of skin tests or in vitro tests, as performed in clinical practice. From this perspective, dogs, neonatal swine, and monkeys might represent particularly interesting species because of their similarity to humans in terms of physiopathology and immune response. Dogs and swine have demonstrated a natural tendency for allergic reactions with similar immunopathogenic consequences and therapeutic interventions as in humans. To the best of our knowledge, these latter species have mostly been used to investigate food allergies so far. Considering a final validation of results established in rodent models in the abovementioned animals, especially for treatment efficacy, might be useful before translation into clinical practice.

From a clinical point of view, anaphylaxis remains a mystery in terms of many aspects. The first is the immunization step: why does one individual develop an allergy but not another? Which route is responsible for the sensitization? What are the key factors associated with this? Animal models bred in pathogen-free environments cannot provide answers to these questions, as the experimental conditions do not reflect the complex and changing environments in which humans develop for many years before noticing symptoms. The second is the endotype of the reaction and the mediators involved. In this regard, animal models might be particularly useful, especially for improving basic molecular knowledge, the analysis of the different pathways involved, and evaluating how receptor polymorphisms and genetic variations can modulate the reaction. For instance, knockout mouse models are useful to study the importance of specific genes in different pathways (IgE/IgG-mediated) and to conduct investigations at the cellular and molecular level (e.g., mast cell activation and the quantification of histamine/PAF release).

Finally, evaluating new treatments for anaphylaxis is particularly challenging in humans as anaphylaxis events are rare and mostly unpredictable, and because treatment must be administered in a life-threatening emergency setting. Controversies also remain as to the use of adjuvant vasopressors [94], such as methylene blue [32] or vasopressin, in humans [33], which can be of major help in patients presenting with refractory anaphylaxis.

## 8. Conclusions

Given the challenges of analyzing the mechanisms involved in human anaphylaxis, animal models have been established to replicate human anaphylaxis and reproduce its pathophysiology. In mouse models, monitoring tools such as rectal temperature, quantitative activity scales, and diarrhea occurrence are routinely used to assess an allergic response. Rats offer an ease of invasive measurements, especially regarding hemodynamic, respiratory, and cerebral oxygenation evaluation. Their use is crucial particularly in assessing treatment efficacy and its effects on cerebral oxygenation, cardiac output, and vascular leakage. Sensitization procedures in animal models depend on various factors, including the administration route, antigen concentration, use of adjuvants, and genetic background, to induce clinical responses that resemble IgE-mediated food and drug allergies in humans. Overall, animal models provide valuable insights into the pathophysiology of anaphylaxis and are essential for testing potential therapies and developing preventive measures for this life-threatening condition.

## Figures and Tables

**Table 1:** Summary of the different monitoring approaches available in mice or rats to evaluate physiological parameters during anaphylaxis experiments.

Animal	Physiological Parameter	Probes for Measurement
Mice/rats	Drop in core body temperature	Rectal temperature
	Level of intestinal mast cells	Occurrence of diarrhea
	Reduced physical activity	Activity scale
Mostly rats	Decreased vascular resistance, fluid loss, and hemodynamic impairment	Tracheal intubation, vein line placement
	Muscular interstitial lactate concentration	PtiO2 and micro-dialysis probes in the quadriceps muscle
	Hemodynamic failure of cerebral oxygenation	Measurements of cerebral cortical blood flow and oxygen partial pressure
	Hemodynamic failure	Perivascular ultrasonic flow probes
	Left-ventricular function impairment	Catheter insertion
	Vascular leakage due to histamine release	Hematocrit measurement

## Data Availability

Not applicable.
